# Diabetogenic liver metastasis from pancreatic cancer: a case report

**DOI:** 10.1186/s40792-022-01582-8

**Published:** 2022-12-28

**Authors:** Sho Kiritani, Yoshihiro Ono, Manabu Takamatsu, Atsushi Oba, Takafumi Sato, Hiromichi Ito, Yosuke Inoue, Yu Takahashi

**Affiliations:** 1 Division of Hepatobiliary and Pancreatic Surgery, Cancer Institute Hospital of the Japanese Foundation for Clinical Research, 3-8-31 Ariake, Koto-Ku, Tokyo, 135-8550 Japan; 2 Department of Pathology, Cancer Institute Hospital of the Japanese Foundation for Cancer Research, Tokyo, Japan

**Keywords:** Pancreatic cancer, Metachronous liver metastasis, New-onset diabetes, Liver resection

## Abstract

**Background:**

Although new-onset diabetes has been described in up to 20% of patients with newly diagnosed pancreatic cancer, reports regarding new-onset diabetes associated with newly developed liver metastasis from pancreatic cancer are limited.

**Case presentation:**

A 60-year-old man was diagnosed with pancreatic tail cancer without impaired glycemic control. A curative-intent distal pancreatectomy with adjuvant S-1 chemotherapy was performed. Two years after surgery, a high HbA1c concentration and solitary liver metastasis were identified on follow-up examination. Two major chemotherapy regimens, gemcitabine/nab-paclitaxel and modified FOLFIRINOX, were sequentially administered to the patient; however, his carbohydrate 19-9 concentration continued to increase. Because the patient’s glycemic control rapidly worsened in synchrony with the tumor growth, insulin therapy was initiated. Although the liver metastasis was refractory to chemotherapy, curative-intent left hepatectomy was performed because only one tumor remained. His impaired glycemic control improved immediately after surgery, and insulin therapy was terminated. When writing this report (2 years after hepatectomy), the patient was alive and recurrence-free.

**Conclusions:**

New-onset diabetes appeared with the progression of metachronous liver metastasis from pancreatic cancer, without recurrence at any other site. The patient’s diabetic state was improved by resection of the liver tumor, and liver metastasis itself was proven to have caused the glucometabolic disorder by increasing insulin resistance.

## Background

Pancreatic cancer is one of the most aggressive and lethal malignant neoplasms. According to a large epidemiological survey, the number of new cases and deaths from pancreatic cancer are almost the same every year, implying that the cure of pancreatic cancer is a challenging issue [1, 2]. Most patients with pancreatic cancer present with nonspecific symptoms until their condition reaches an advanced stage that makes curative-intent surgery difficult to achieve [3].

Multiple studies have identified diabetes as a risk factor for pancreatic cancer. Recent studies have shown a diabetes prevalence of > 40% in patients with pancreatic cancer [4–7]. A subset of these patients develops diabetes several months to years before cancer diagnosis. Patients with new-onset pancreatogenic diabetes account for at least 20% of patients with newly diagnosed pancreatic cancer, and they may have a localized, surgically resectable tumor at the time of diagnosis, providing a window of opportunity for early cancer detection and treatment [4, 7–9].

Although previous reports have suggested that new-onset diabetes can improve after pancreatectomy for primary pancreatic cancer, little is known about the relationship between metachronous liver metastasis from pancreatic cancer and new-onset diabetes [9, 10]. We herein describe a patient who developed metachronous liver metastasis from pancreatic cancer with acute impaired glycemic control and achieved successful insulin withdrawal after curative hepatectomy and long-term recurrence-free survival.

## Case presentation

**Fig. 1:**
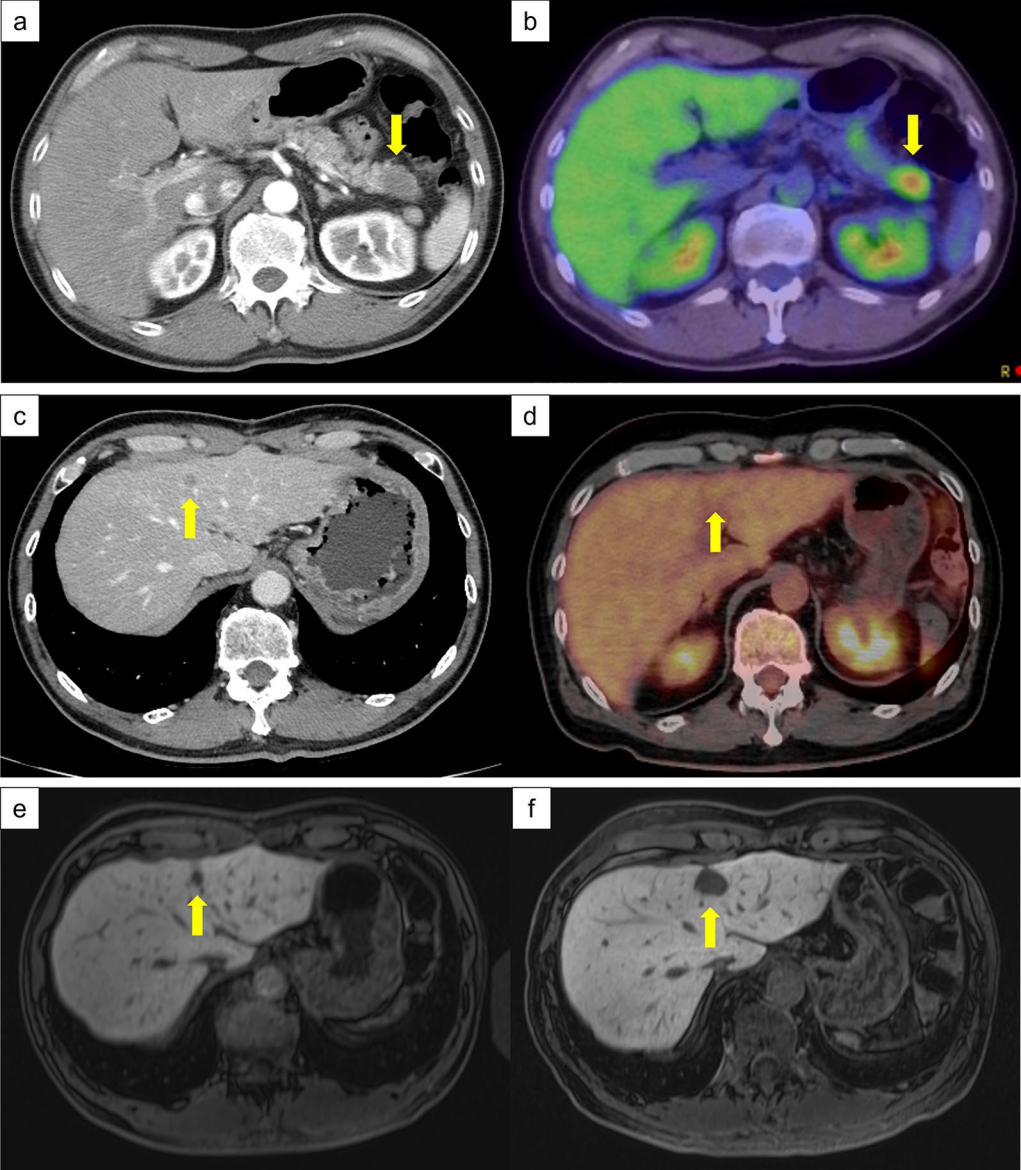
HYPERLINK "sps:id::fig1||locator::gr1||MediaObject::0" Imaging study for pancreatic cancer and liver metastasis. **a** Late arterial phase of dCECT revealed a hypovascular lesion at the pancreatic tail. The tumor diameter was 3.0 cm. **b** Positron emission tomography reveals a high standardized uptake value (maximum 6.6). **c** Two years after curative pancreatic resection, a small hypovascular liver lesion was identified in segment 4. The tumor diameter was 1.2 cm. **d** Positron emission tomography at the diagnosis of liver metastasis reveals a standardized uptake value of 3.3, which was equivalent to normal liver parenchyma. **e**, **f** Change that occurred from before to after chemotherapy for liver metastasis. Although the tumor diameter increased to 2.5 cm, the number of tumors did not change. *dCECT* dynamic contrast-enhanced computed tomography

A 60-year-old man was referred to our hospital with suspected pancreatic tail cancer based on a high carbohydrate antigen 19-9 (CA19-9) concentration found during a medical checkup. He was a past smoker (Brinkman Index, 800) and drinker (20 g/day of ethanol for 20 years). He had no remarkable past illnesses, including diabetes, and no familial history of pancreatic cancer. His body mass index was 20.8 kg/m^2^. The patient had no weight loss events. Dynamic contrast-enhanced computed tomography (dCECT) at our hospital revealed a hypovascular lesion in the pancreatic tail (Fig. 1a). Adenocarcinoma was confirmed using endoscopic ultrasonographic fine-needle aspiration. His CA19-9 concentration at our hospital was 286.8 U/mL, and his HbA1c concentration was 5.6%. dCECT revealed that the tumor was anatomically resectable. No synchronous liver metastasis was identified by gadolinium ethoxybenzyl diethylenetriamine pentaacetic acid-enhanced magnetic resonance imaging (MRI). Positron emission tomography revealed a high standardized uptake value (maximum 6.6; Fig. 1b). Thus, the patient underwent distal pancreatectomy and regional lymph node dissection in accordance with radical antegrade modular pancreatosplenectomy [11]. In this surgery, the pancreatic parenchyma was cut just above the superior mesenteric vein using tri-stapler technology. The operative time was 251 min, and extra blood loss was 720 mL. Although he developed a clinically relevant postoperative pancreatic fistula, it improved without any invasive intervention, and he was discharged on postoperative day 40. Pathological examination revealed a 36 × 18 × 17-mm moderately differentiated tubular adenocarcinoma. No lymph node metastasis was identified (0/27). No microscopic lymphatic invasion was observed, but microscopic venous invasion was observed. The cancer slightly invaded the extrapancreatic connective tissue. The pancreatic cut and dissected margins were negative. Postoperative staging was stage IB (T2N0M0) according to the Union for International Cancer Control 8th edition. Adjuvant S-1 chemotherapy (120 mg/body/day) was administered for 6 months after surgery.

**Fig. 2:**
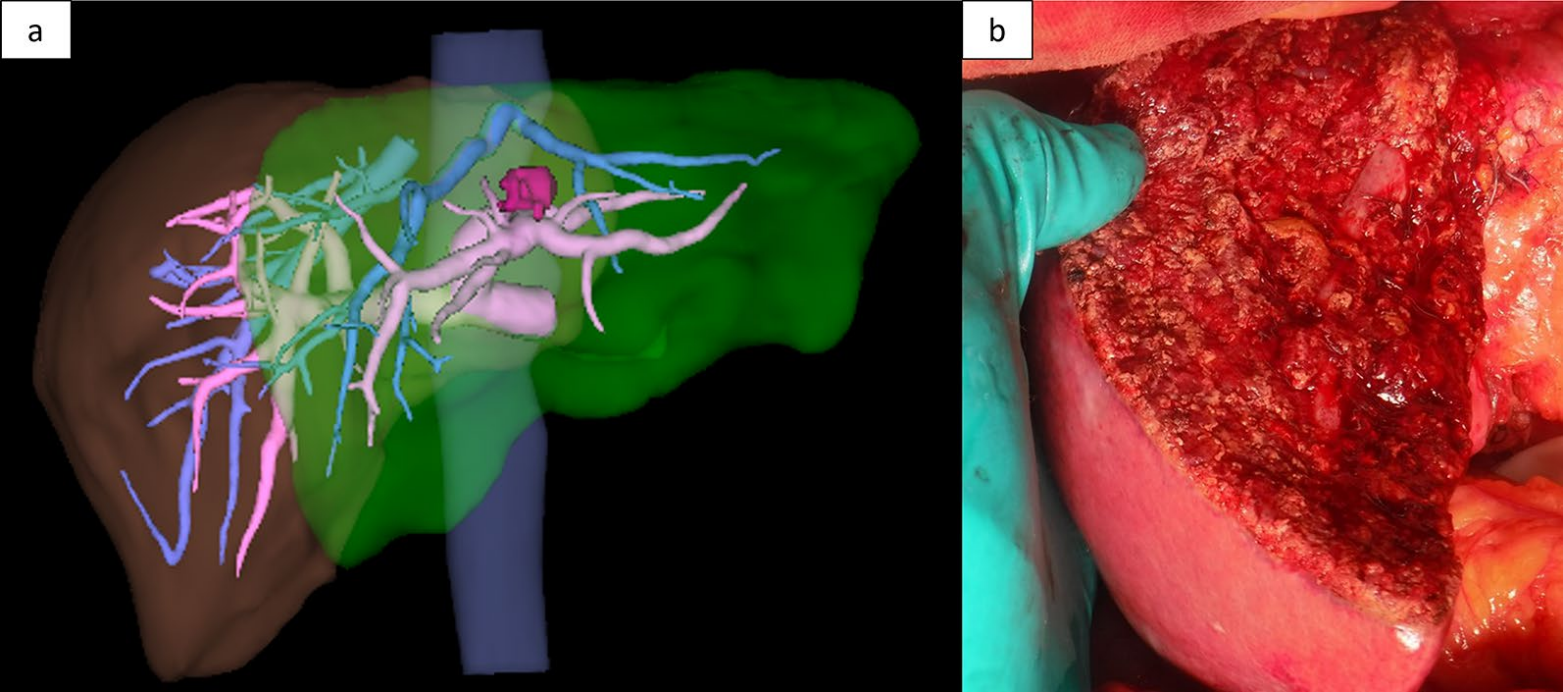
**a** Preoperative three-dimensional simulation. The pink mass is a liver metastasis, which was close to the umbilical portion. The estimated left hepatectomy region is colored green. **b** Intraoperative photograph after left hepatectomy

**Fig. 3:**
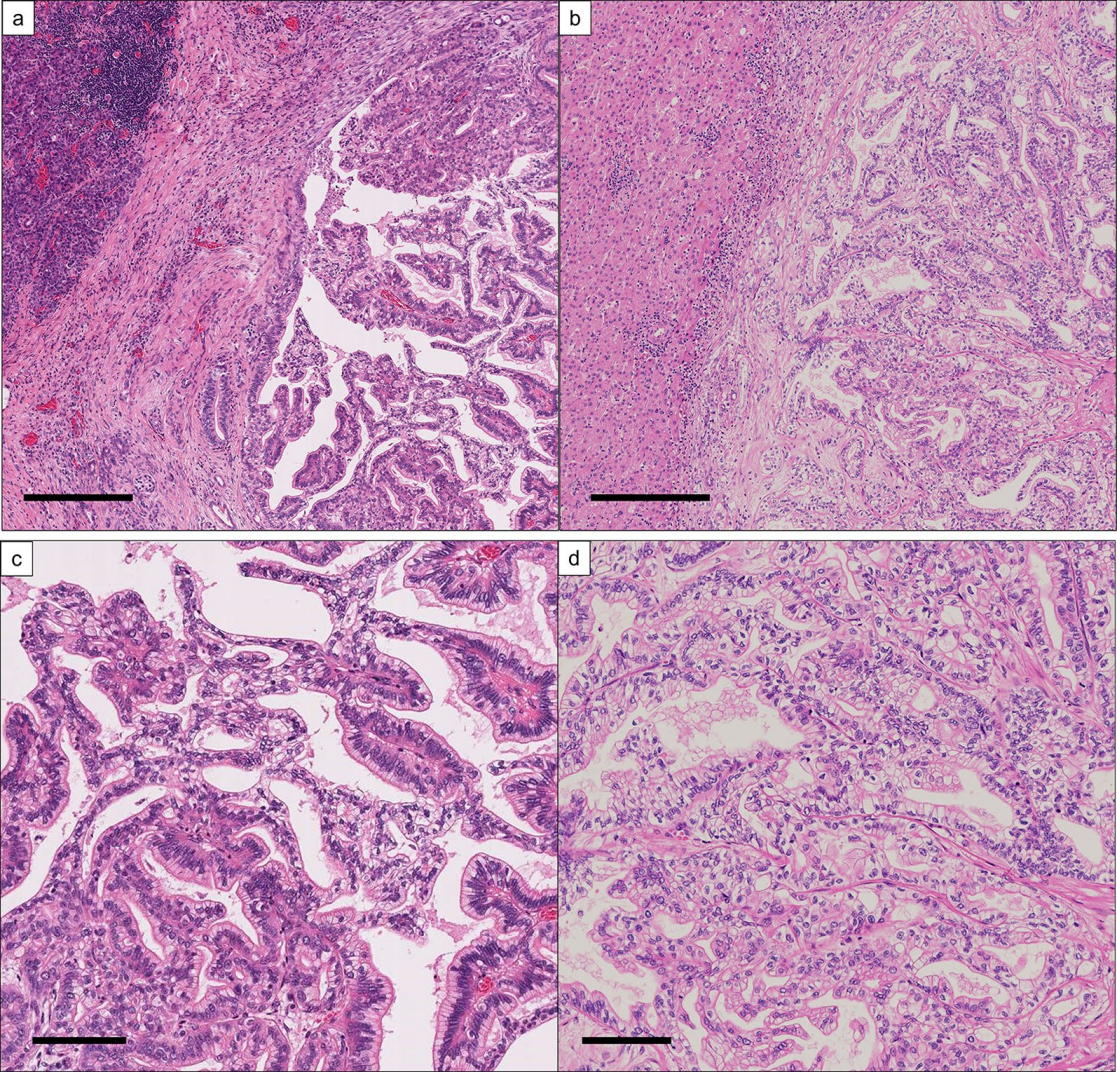
Histologic appearances of **a** the primary pancreatic cancer and **b** its liver metastasis. Enlarged views are shown in **c** and **d**, respectively. The cancer cells have round nuclei with clear cytoplasm and show a tubulopapillary architecture. Scale bars, 300 µm (low-power field), 100 µm (high-power field)

Two years after the initial pancreatectomy, a solitary hypovascular lesion was identified in liver segment 4 on follow-up dCECT (Fig. 1c). This was considered liver metastasis from pancreatic cancer in conjunction with the MRI results. The CA19-9 concentration was slightly elevated at 44.5 U/mL. However, the tumor did not show a significant standardized uptake value on positron emission tomography (Fig. 1d). Gemcitabine 1000 mg/m^2^ + nab-paclitaxel 125 mg/m^2^ was administered on day 1, 8, and 15 every 4 weeks for the liver metastasis. Dexamethasone (6.6 mg) was administered as a premedication. After 14 cycles of gemcitabine/nab-paclitaxel, the CA19-9 level, which had fallen to the normal range, became elevated again (660.7 U/mL) and the tumor had increased in size (from 10 to 15 mm in diameter); therefore, the chemotherapy was converted to a modified FOLFIRINOX regimen (5-fluorouracil 2400 mg/m^2^, levoleucovorin 200 mg/m^2^, oxaliplatin 85 mg/m^2^, and irinotecan 150 mg/m^2^) as second-line therapy, which was administered triweekly. Steroid use as a premedication remained unchanged (6.6 mg dexamethasone). However, the CA19-9 concentration further increased to 3130.0 U/mL after nine cycles of FOLFIRINOX chemotherapy. The tumor diameter increased to 25 mm; however, no further metastases developed. The MRI findings before and after chemotherapy are summarized in Fig. 1e, f. The number of tumors did not change (i.e., the patient did not develop more than one tumor), the tumor was resectable, chemotherapy was minimally effective, and the patient’s general status was well maintained; therefore, we decided to perform surgical resection at this time after a multidisciplinary team conference. Left hepatectomy and cholecystectomy were also performed. Preoperative three-dimensional simulation and intraoperative photographs are shown in Fig. 2. The operation time was 239 min and the blood loss was 480 mL. Pathological examination revealed that the tumor was a moderately differentiated adenocarcinoma compatible with metastasis from pancreatic cancer (Fig. 3a–d). On hematoxylin and eosin staining, there was little heterogeneity between the primary and metastatic lesions. No microscopic vessel invasion (portal, venous, arterial, or bile) was found. The surgical margins were negative. Adjuvant therapy was not administered after the second surgery. The patient was alive with no recurrence 2 years after hepatectomy.

**Fig. 4:**
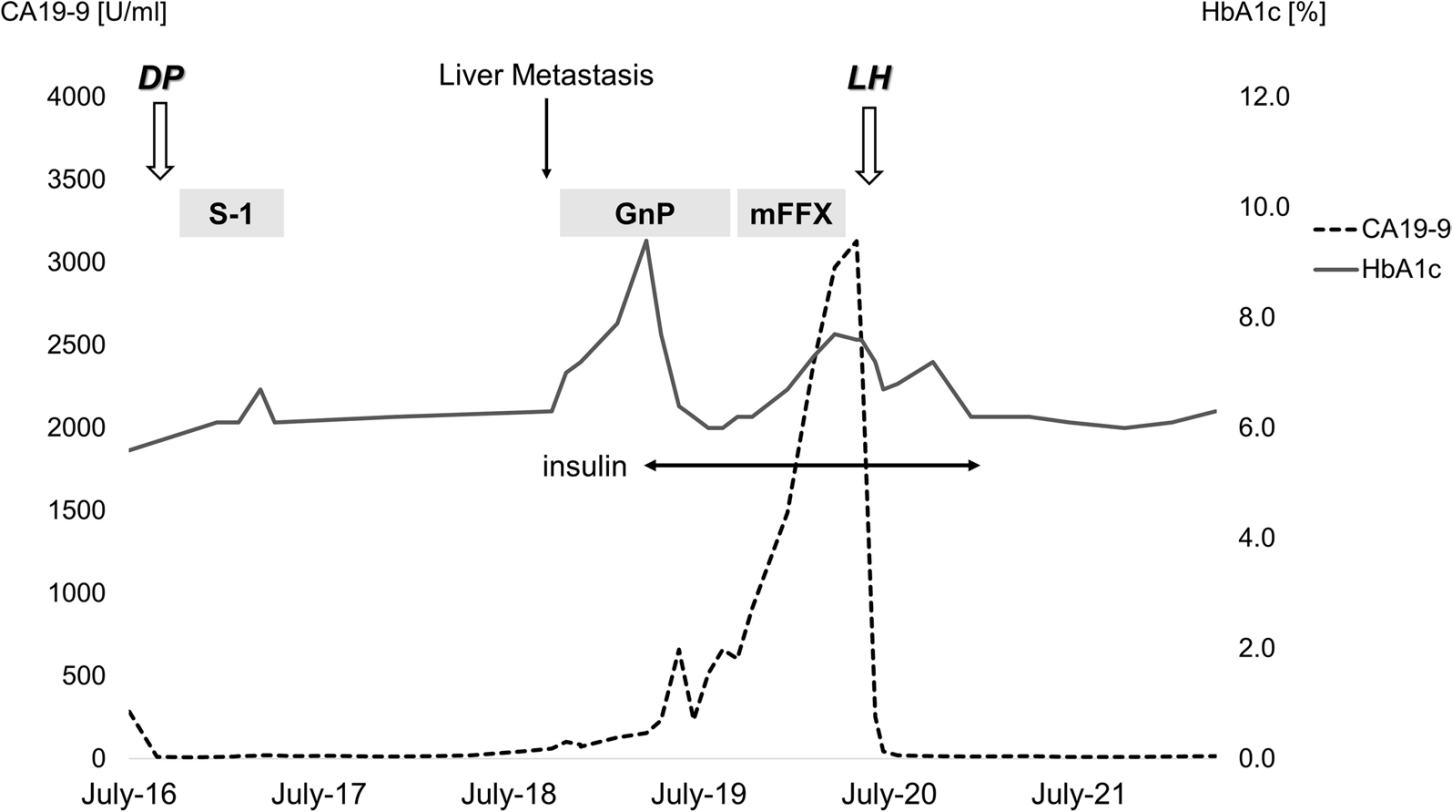
Summary of patient’s clinical course. *DP* distal pancreatectomy, *LH* left hepatectomy, *GnP* gemcitabine and nab-paclitaxel, *mFFX* modified FOLFIRINOX, *LM* liver metastasis, *CA19-9* carbohydrate antigen 19-9

At the time of diagnosis of liver metastasis, the HbA1c level was elevated to 7.2%. Thereafter, this value increased rapidly; 6 months later, it increased to 10.0%, and insulin therapy was initiated. The serum C-peptide concentration was 3.65 ng/mL at that time. Although the HbA1c concentration decreased after insulin induction, it increased again in parallel with the increase in CA19-9 concentration. Finally, 15 units of regular insulin and 18 units of long-acting insulin were administered for preoperative glycemic control. After curative hepatectomy, the patient’s glycemic control improved immediately. The patient started oral intake and insulin injection at the same amount as before on postoperative day 2. Blood glucose levels fluctuated between 100 and 150 mg/dL. Therefore, the dose of insulin was reduced. At discharge, regular insulin was terminated. Furthermore, insulin therapy was completely terminated 2 months after the surgery. The clinical course of the patient is summarized in Fig. 4.

## Discussion

Herein, we report a unique and informative case of a patient who developed metachronous liver metastasis from pancreatic cancer and simultaneous impaired glycemic control that had not been present at the initial development of pancreatic cancer. Glycemic control improved with the initiation of insulin therapy, but worsened again with an increase in CA19-9 concentration. Although this tumor marker level increased and the tumor gradually enlarged after chemotherapy, the number of liver metastases did not exceed one, and no extrahepatic lesions emerged. Thus, curative-intent liver resection was performed, and the patient achieved 2-year recurrence-free survival. Glycemic control immediately improved after the liver resection, and the patient was able to discontinue insulin therapy.

The complex relationship between diabetes and cancers, such as colorectal, hepatic, and pancreatic cancers, has long been recognized [7, 12]. Pre-existing diabetes has been reported to account for 40–50% of patients diagnosed with pancreatic cancer, and half of these patients are classified as having new-onset diabetes (defined as type 3C diabetes according to the American Diabetes Association classification) [13–16]. A causal relationship between pancreatic cancer and new-onset diabetes is supported by the resolution of diabetes after surgical resection of the tumor in > 50% of patients [7, 17]. In contrast, improvement in glycemic control may be associated with the surgical procedure itself because it has also been demonstrated that pancreatoduodenectomy similarly improves diabetes in patients with or without pancreatic cancer [18, 19]. Therefore, several explanations exist regarding the mechanism of new-onset diabetes in relation to pancreatic cancer. In the present case, pancreatic resection was not performed during treatment of the metachronous liver metastasis, which provides supporting evidence that the acute impairment of glycemic control was caused by the metastatic lesion. Metachronous distant metastasis from pancreatic cancer can create a “diabetogenic” state. New-onset diabetes is considered to be characterized by impaired insulin secretion and insulin resistance. The C-peptide concentration in the patient in this report was maintained at > 3.0 ng/mL, indicating that his impaired glycemic control was derived from insulin resistance. Although only a few studies have been substantiated to date, it is essential to search for systemic mediators (macrophage migration inhibitory factor, adrenomedullin, and dipeptidyl peptidase 4) [20–22]. Further comprehensive research is warranted to elucidate these detailed mechanisms.

Steroids can be a cause of newly diagnosed diabetes. A previous study indicated that > 20 mg prednisolone or equivalent daily use was associated with a high risk of developing steroid-induced hyperglycemia [23]. However, the newly diagnosed hyperglycemia in this case was not considered to be derived only from steroid administration for the following reasons: first, the total dosage of steroids was lower than that of high-risk patients. Second, the HbA1c level increased before the induction of chemotherapy (see Fig. 4). Third, although the dexamethasone dose decreased from once a week to once every 3 weeks when the chemotherapy regimen changed from gemcitabine + nab-paclitaxel to modified FOLFIRINOX, HbA1c levels increased. The second factor to consider was post-pancreatic diabetes. A previous systematic review revealed that the incidence of new-onset diabetes after distal pancreatectomy is 3–40% [24]. The review also reported that the median time to the onset of diabetes was early, within 1 year after surgery. In this case, the HbA1c level had been stable within the normal range (5.8–6.3%) for approximately 2 years after the initial distal pancreatectomy (see Fig. 4), and it abruptly worsened when liver metastasis was diagnosed. Thus, this patient was unlikely to have developed new-onset diabetes after pancreatectomy.

In this case, the growth of the liver metastasis and impaired glycemic control progressed in parallel. Previous studies have suggested that poor glycemic control is associated with failed neoadjuvant chemotherapy or surgery [25, 26]. It is difficult to determine whether a tumor that responds poorly to chemotherapy worsens glycemic control or poor glycemic control induces chemotherapy intolerance. In the present case, however, after initiation of insulin, the HbA1c concentration decreased, and the CA19-9 concentration also decreased (Fig. 4). Thus, good glycemic control might temporarily improve tumor susceptibility to chemotherapy. Indeed, it also seems rational to consider that exacerbation of tumor progression would further worsen glycemic control through the emission of biochemical substances from the tumor. Further research will reveal the relationship between glycemic control and tumor biology. Notably, CA19-9 levels have been reported to be closely associated with HbA1c levels [27].

Impaired glycemic control was not observed during the treatment of the primary pancreatic cancer, but instead at the metachronous liver metastasis. This seems to reflect the biological heterogeneity between primary pancreatic cancer and metachronous liver metastases. This can also be indicated by the fact that the standardized uptake value in positron examination was different between primary pancreatic cancer and metachronous liver metastasis. Intratumor heterogeneity has been identified in recent genetic studies [28]. Metastasis is a multistep process consisting of local invasion, intravasation, survival in circulation, extravasation, and distant colonization [29, 30]. Two prominent models of metastasis have been considered: the linear model and the parallel progression model [31]. In the linear progression model, metastasis is seeded at the late stage of tumor progression, resulting in minimal genetic divergence between the primary tumor and its metastases. Conversely, in the parallel progression model, metastases are seeded early in tumor progression, and high levels of genetic divergence are expected between the primary tumor and its metastases [32, 33]. In this case, the influence of metachronous liver metastasis on impaired glycemic control was overtly different from that of primary pancreatic cancer; therefore, the metastatic pattern was considered to be consistent with the parallel progression model.

## Conclusions

Herein, we present a case of a patient who showed acute impaired glycemic control with metachronous liver metastasis from pancreatic cancer. This case has further implications regarding the correlation between chemoresistance and poor glycemic control, heterogeneity of metastasis from pancreatic cancer, and the complex indications for metastasectomy.

## Data Availability

All data generated or analyzed during this study are included in this article. Further inquiries can be directed to the corresponding author.

## Abbreviations

CA19-9: Carbohydrate antigen 19-9
dCECT: Dynamic contrast-enhanced computed tomography
MRI: Magnetic resonance imaging
